# Tuberculosis in Children

**Published:** 1899-02-11

**Authors:** 


### TUBERCULOSIS IN CHILDREN.

In an able article in the *Lancet* Dr. Leonard Guthrie controverts the idea which has been maintained with so much force by Sir Richard Thorne and others as to the increase of tabes mesenterica in children under one year of age, and also of tuberculous meningitis, and as to the relation of these diseases to the consumption of milk and to the reception therefrom of tuberculous infection into the alimentary canal. As is well known, it has been shown by experiment that guinea-pigs when fed on tuberculous material soon develop tabes mesenterica, and also that cows with tuberculous udders give tuberculous milk, and the inference has been drawn that infants, who are presumably fed chiefly on milk, become tuberculous by drinking it. Dr. Carr, however, from his records of post-mortem examinations at the Victoria Hospital for Children, shows that tabes mesenterica is rare in children under two years of age and almost unknown in those under one year of age, and while he regards the increase of meningitis as being due to increasing correctness of diagnosis, he looks upon increasing incorrectness of diagnosis in regard to tabes mesenterica as explaining the alleged increase in that disease among infants. To arrive at some conclusions as to this matter Dr. L. Guthrie has tabulated his notes of post-mortem examinations made on children dying from tuberculosis in the Children's Hospital, Paddington Green, during eight years, and he comes to the conclusion that (1) thoracic tuberculosis in children is more common than abdominal in the proportion of three to two; (2) tabes mesenterica as a cause of death in young children is practically unknown; (3) the preponderance of thoracic over abdominal tuberculosis is not necessarily and solely due to the direct entry of bacilli into the air passages; (4) on the other hand, primary infection through the alimentary canal does not prove that food has been the sole source of evil; therefore tuberculosis in children is not likely to be materially checked by purification of milk supply alone; (5) the alleged increase of tuberculous meningitis of late years is probably due to pulmonary tuberculosis set up by severe epidemics of measles. Clearly it is well to hear both sides.

